# Diet quality and physical activity affect metabolic dysfunction-associated steatotic liver disease, metabolic dysfunction and etiology-associated steatohepatitis, and compensated advanced chronic liver disease among United States adults: NHANES 2017–2020

**DOI:** 10.3389/fnut.2024.1505970

**Published:** 2025-01-08

**Authors:** Peng Wang, Bingxin Xia, Shuang Wang

**Affiliations:** ^1^Department of Geriatrics, The People’s Hospital of Changshou, Chongqing, China; ^2^Department of Geriatrics, The Second Affiliated Hospital of Chongqing Medical University, Chongqing, China

**Keywords:** MASLD, MetALD, cACLD, physical activity, diet quality

## Abstract

**Background and aim:**

Clinical data on the prevalence of metabolic dysfunction-associated steatotic liver disease (MASLD) and metabolic dysfunction and etiology-associated steatohepatitis (MetALD) in a multi-ethnic U.S. population are limited. Additionally, the impact of physical activity (PA) and diet quality (DQ) on the risk of MASLD, MetALD, and compensated advanced chronic liver disease (cACLD) remains unclear. This study aimed to investigate the associations of PA and diet quality with the risks of MASLD, MetALD, and cACLD.

**Methods and results:**

This cross-sectional study analyzed data from 7,125 participants in the National Health and Nutrition Examination Survey (NHANES) 2017–2020. Diet quality was assessed using the Healthy Eating Index-2015 (HEI-2015). PA was assessed based on the 2020 WHO Physical Activity Guidelines, with participants reporting the intensity, frequency, and duration of their activities over the past 7 days. MASLD and MetALD were diagnosed based on clinical criteria, and cACLD was defined by advanced liver fibrosis. Bivariate and multivariable logistic regression models were used to assess associations between PA, diet quality, and liver disease outcomes. The prevalence of MASLD and MetALD was 35.07 and 21.46%, respectively. HQD was associated with significantly lower risks of MASLD (OR: 0.49, 95% CI: 0.38–0.62) and MetALD (OR: 0.45, 95% CI: 0.36–0.56). High PA levels were linked to reduced risks of MASLD (OR: 0.47, 95% CI: 0.38–0.58) and MetALD (OR: 0.53, 95% CI: 0.39–0.72). The lowest risks for both MASLD and MetALD were observed in highly active participants with an HQD (MASLD OR: 0.41, 95% CI: 0.32–0.53; MetALD OR: 0.54, 95% CI: 0.41–0.71). Significant interactions were observed between PA, HQD, and age, BMI, and SES, which further reduced the risks of MASLD and MetALD. For cACLD, both increased PA and HQD were associated with reduced risk. Compared to non-high-activity participants with a non-HQD, physically active participants with an HQD had the lowest risk of cACLD (OR: 0.44, 95% CI: 0.24–0.82).

**Conclusion:**

High proportions of the US population have MASLD or MetALD. HQD and high PA levels were associated with lower risks of MASLD, MetALD, and cACLD.

## 1 Introduction

Non-alcoholic fatty liver disease (NAFLD), affecting approximately 25% of the global population, poses a significant challenge in the management of chronic liver diseases (1). However, the traditional diagnostic framework for NAFLD, which relies on exclusion criteria, inadequately represents the underlying pathophysiology, particularly its association with metabolic dysregulation. To address this, the term “metabolic dysfunction-associated fatty liver disease” (MAFLD) was introduced to describe a spectrum of liver conditions linked to metabolic dysregulation (2, 3). This shift marked an important step toward aligning the terminology with the disease’s metabolic origins. Despite this improvement, the term MAFLD was still seen as somewhat narrow and potentially stigmatizing, as it did not fully capture the heterogeneity of liver diseases with steatosis. In response to these concerns, the nomenclature evolved further with the introduction of “steatotic liver disease” (SLD), a broader classification that replaces both NAFLD and MAFLD (4). SLD categorizes patients based on the presence or absence of cardiometabolic risk factors (CMRFs), acknowledging the diverse metabolic contributors to liver disease while avoiding the restrictive focus on metabolic dysfunction alone. Within this framework, patients with CMRFs are classified as having “metabolic dysfunction-associated steatotic liver disease” (MASLD), highlighting steatosis primarily driven by metabolic factors. Conversely, patients with moderate alcohol intake or steatosis resulting from drugs, monogenic diseases, or other combined etiologies are categorized under “metabolic dysfunction and etiology-associated steatohepatitis” (MetALD) (5). These classifications refine diagnostic accuracy and better capture the diverse drivers and progression pathways of steatotic liver diseases.

A critical outcome of these diseases is the progression to advanced stages such as compensated advanced chronic liver disease (cACLD) (6). cACLD is characterized by significant fibrosis where the liver retains its essential functions but is at high risk for further decompensation and liver-related complications (7). Clinically, cACLD marks a pivotal stage in chronic liver disease, as interventions at this point can prevent irreversible damage and improve prognosis (8). However, there is limited research on the modifiable lifestyle factors, such as physical activity (PA) and diet quality (DQ), in mitigating cACLD risk.

Understanding MASLD, MetALD, and cACLD is essential given the increasing prevalence of these conditions, driven largely by global lifestyle trends. Regular PA improves metabolic health, reduces liver fat, and mitigates inflammation, all of which are critical in the pathophysiology of liver diseases (9, 10). Similarly, DQ directly influences liver health, with unhealthy diets exacerbating steatosis and fibrosis progression (11, 12). Although PA and DQ are recognized as important, limited research has explored their combined impact on MASLD, MetALD, and cACLD.

Therefore, this study aimed to analyze data from the National Health and Nutrition Examination Survey (NHANES) 2017–2020 to investigate the associations of PA and DQ with the risks of MASLD, MetALD, and cACLD.

## 2 Materials and methods

### 2.1 Study design and participants

This study used data from the 2017–2020 cycle of the NHANES, a comprehensive cross-sectional survey program conducted in the US. The NHANES evaluates various health and nutritional aspects of the general US population. The participants provided self-reported information through structured household interviews covering demographic details, medical history, dietary habits, and PA levels. Additionally, physical examinations, including anthropometric measurements and blood sample collection, were conducted at mobile examination centers (13). The present study analyzed publicly accessible data from NHANES 2017–2020, including data collection procedures, analytical guidelines, and complete datasets (14).

### 2.2 Study sample

**Figure 1:**
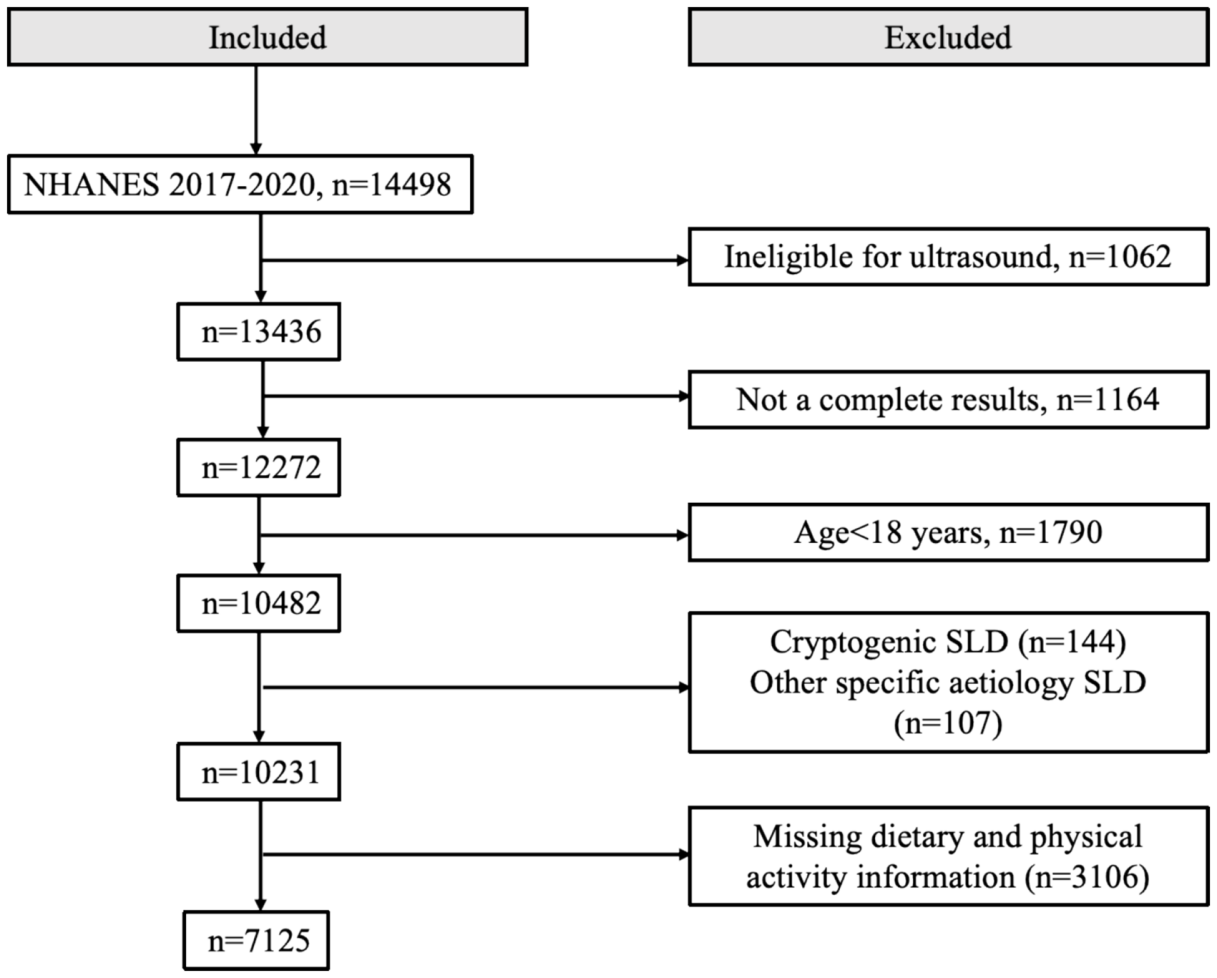
Flowchart of the selection in this study.

This study included individuals who were 18 years of age or older and had available data on ultrasonographic determination of hepatic steatosis. The exclusion criteria were as follows: (1) liver diseases associated with other factors, such as positive hepatitis B surface antigen or positive hepatitis C antibody, or severe alcohol consumption; (2) missing data on dietary intake or physical activity; (3) missing data for assessing MASLD and MetALD; (4) missing data on smoking status, hypertension, dyslipidemia, diabetes, and other relevant conditions. As a result, the final study sample consisted of 7,125 adults with complete data. The specific selection process is shown in Figure 1.

### 2.3 Diagnostic criteria and definition of groups

#### 2.3.1 MASLD and MetALD

Based on the recent Delphi consensus, SLD was defined as the presence of evidence of liver steatosis from ultrasound finding (15). The criteria for diagnosing liver steatosis included a controlled attenuation parameter (CAP) threshold of 285 dB/m, as previously established in the literature (16). Participants with evidence of liver steatosis who met this CAP threshold were classified as having SLD. Within participants with SLD, metabolic dysfunction was diagnosed based on the presence of one or more of the following criteria: body mass index (BMI) ≥25 kg/m^2^ or a waist circumference (WC) ≥94 cm (men) and ≥80 cm (women); fasting plasma glucose ≥100 mg/dL, or hemoglobin A1c (HbA1c) ≥5.7%, a previous diagnosis of type 2 diabetes (T2D), or undergoing treatment for T2D; blood pressure ≥ 130/85 mmHg, or receiving treatment for hypertension; triglyceride level ≥ 150 mg/dL, or on lipid-lowering therapy; high-density lipoprotein cholesterol level < 40 mg/dL (men) or <50 mg/dL (women), or on lipid-lowering therapy. Patients without viral hepatitis or significant alcohol consumption were diagnosed with MASLD. In contrast, patients with MASLD who consumed >140–350 g (women) and >210–420 g (men) of alcohol per week or who had drug-induced or monogenic disease-related steatosis were diagnosed with MetALD (17, 18).

### 2.4 Definition of liver steatosis and fibrosis

A controlled attenuation parameter of 285 dB/m was used as the threshold to diagnose liver steatosis, as described previously. Regarding liver fibrosis, a liver stiffness measurement ≥10 kPa indicated MASLD-related compensated advanced chronic liver disease (cACLD), analogous to the cutoffs used for significant liver fibrosis and high-risk metabolic-associated steatohepatitis (7, 19).

### 2.5 Demographic variables

The NHANES questionnaire collected demographic information, including age, sex, race, education level, and household income. Race was categorized as non-Hispanic white, non-Hispanic black, non-Hispanic Asian, Mexican-American, and other or multiracial. Education level was classified as less than high school graduate, high school graduate or equivalent, and college education or higher. Household income levels were categorized as low, middle, and high (poverty income ratio [PIR] <1.30, 1.30≤ to <3.50, and ≥3.50, respectively). For each participant, self-reported education and household income served as indicators of socioeconomic status (SES) (20).

### 2.6 Lifestyle variables

Lifestyle factors assessed included smoking history, physical activity (PA), and self-reported dietary intake. Smoking status was categorized as current (≥100 lifetime cigarettes and currently smoke), former (≥100 cigarettes and have quit), and never-smokers (<100 lifetime cigarettes) (21). The Healthy Eating Index (HEI) is a measure of diet quality, assessing how closely a set of foods aligns with the Dietary Guidelines for Americans (22). The HEI-2015 includes 13 components, evaluating factors such as the intake of fruits, vegetables, whole grains, dairy, protein foods, saturated fat, and added sugars. These components replace the “empty calories” used in HEI-2010, providing a more precise evaluation of diet quality (23). HEI-2015 scores range from 0 to 100, with higher scores indicating better diet quality. To calculate the HEI-2015 score, we used the total nutrient intake data from the first 24-h dietary recall for each participant. The NHANES diet-related variables used for this calculation, detailed in Supplementary Table S1, include total energy intake (DR1TOT_C), kilocalories (DR1TKCAL), protein (DR1T_PROT), fat (DR1T_FAT), saturated fat (DR1T_SFAT), carbohydrates (DR1T_CARB), sugar (DR1T_SUGR), and fiber (DR1T_FIBE), among others. These data were used to compute the individual components of the HEI-2015, which were then aggregated into an overall diet quality score. The HEI-2015 scores were grouped into tertiles for further analysis: high (>55.67), borderline (42.25–55.67), and low (<42.25). For PA assessment, participants were asked about the intensity, frequency, and duration of their activities over the past 7 days. According to the 2020 WHO Physical Activity Guidelines (24), 1 min of vigorous-intensity activity is considered equivalent to 2 min of moderate-intensity activity. The guidelines recommend that adults engage in at least 150 min of moderate-intensity physical activity per week (or 75 min of vigorous-intensity, or an equivalent combination), with greater benefits observed with more than 300 min. In this study, total physical activity minutes per week were calculated by adding the time spent on moderate-intensity physical activity to twice the time spent on vigorous-intensity physical activity, accounting for the intensity of the activity. Participants were then categorized into three groups based on their total physical activity volume: low active (physical activity volume < 150 min per week), moderate active (≥150 min but <300 min per week), and high active (≥300 min per week). The NHANES physical activity-related variables used for assessment, detailed in Supplementary Table S2, include PAQ605, PAQ620, and PAQ665, which provided data on participants’ weekly activity levels and allowed for the calculation of total PA volume and categorization based on intensity and frequency.

### 2.7 Anthropometric variables

Anthropometric measurements included height, weight, and WC, obtained during physical examinations. BMI was calculated as weight divided by height squared (kg/m^2^) and categorized as normal or underweight (≤25), overweight (>25 to <30), and obese (≥30) (25).

### 2.8 Clinical and biochemical variables

Clinical comorbidities included hypertension, prediabetes, diabetes mellitus, and atherosclerotic cardiovascular diseases such as coronary artery disease and cerebrovascular diseases. Blood samples were analyzed for plasma fasting glucose, fasting insulin, blood lipids, liver function tests, and hypersensitive C-reactive protein (hs-CRP) using enzymatic methods. Hemoglobin A1c (HbA1c) levels were measured using high-performance liquid chromatography.

### 2.9 Power analysis

*A priori* power analysis was conducted to determine the minimum sample size required to detect significant associations between PA, diet quality DQ, and the risks of MASLD, MetALD, and cACLD. Based on previous studies and pilot data, we estimated that an effect size (Cohen’s *d*) of 0.2 would be clinically relevant for detecting differences in PA and DQ levels between individuals with and without these conditions (26). Using an *α* level of 0.05 and a power of 0.80, the required sample size was calculated using G*Power software (27). The analysis indicated that a minimum of 1,000 participants would be needed to detect significant differences. Our study included 7,125 participants, which exceeds the required sample size, thereby ensuring sufficient power to detect meaningful associations.

### 2.10 Statistical analysis

The NHANES uses detailed, multistage probability sampling to represent the US civilian population, with oversampling of certain subgroups to improve estimation accuracy. This analysis incorporated NHANES weights to compensate for survey design complexities, non-responses, and post-stratification, ensuring that our findings are representative of a broader population.

Chi-square tests were used to assess the distribution of categorical variables including age group, sex, race, education level, comorbidities, PA, and HEI-2015 tertiles among patients with MASLD, MetALD, and cACLD. Student’s *t*-tests were used to compare continuous variables, including blood biochemical and physical parameters.

Bivariate and multivariable logistic regression analyses were conducted to calculate odds ratios (ORs) and 95% confidence intervals (CIs) for associations of HEI-2015, PA, education level, and PIR with MASLD, MetALD, and cACLD risks. The inclusion of education level and PIR in these analyses was informed by evidence indicating that socioeconomic factors may influence the relationships between lifestyle factors (e.g., PA and DQ) and liver disease outcomes (28). Subgroup analyses stratified by age, gender, race, education level, PIR, and BMI were performed to explore whether the associations between PA, diet quality, and liver disease outcomes varied across population subgroups. Interaction terms were incorporated into the multivariable logistic regression models to evaluate potential statistical interactions between stratifying variables and exposures. Potential confounders, including age, sex, race, education level, PIR, smoking status, PA, and HEI-2015 scores, were included as covariates in the multivariable logistic regression models based on prior literature and clinical relevance (29).

Two-tailed *p* < 0.05 was considered statistically significant. All analyses were performed using R software version 4.2.3.

### 2.11 Ethics statement

The authors take full responsibility for all aspects of the work, ensuring that any questions concerning the accuracy or integrity of any part of the work are properly investigated and resolved. The study was conducted in accordance with the Declaration of Helsinki (as revised in 2013).

## 3 Results

### 3.1 Prevalence

The MASLD group exhibited a higher prevalence of older age groups, with 32.53 and 29.45% aged 50–64 and ≥65 years, respectively. The MetALD group showed a more even age distribution, with a notable decrease in prevalence among those aged ≥65 years (13.05%). Compared with that in women, the prevalence of MASLD (55.48%) and MetALD (52.01%) was higher in men. The non-Hispanic white individuals had the highest percentages across all groups (Table 1).

**Table 1:** Characteristics of individuals with non-SLD, MASLD, and MetALD in the NHANES study, United States, 2017–2020 (*n* = 7,125).

	Non-SLD Weighted mean or percentage (95% CI) *n* = 3,025	MASLD Weighted mean or percentage (95% CI) *n* = 2,555	MetALD Weighted mean or percentage (95% CI) *n* = 1,545	*p*-value
Age (years)				<0.001
18–34 (*n* = 1,698)	41.13(38.55,43.72)	15.93(14.85,17.01)	27.09(23.93,30.25)	
35–49 (*n* = 1861)	23.63(21.74,25.51)	22.09(19.94,24.23)	27.88(24.31,31.46)	
50–64 (*n* = 1,551)	19.75(17.80,21.69)	32.53(30.01,35.06)	31.98(28.38,35.57)	
≥65 (*n* = 2015)	15.49(13.85,17.13)	29.45(27.05,31.85)	13.05(10.70,15.39)	
Gender				<0.001
Female (*n* = 3,599)	56.66(54.70,58.63)	44.52(42.11,46.93)	47.99(45.96,50.01)	
Male (*n* = 3,526)	43.34(41.37,45.30)	55.48(53.07,57.89)	52.01(49.99,54.04)	
Race				<0.001
NH-White (*n* = 2,399)	62.92(59.10,66.75)	62.40(58.44,66.36)	60.85(56.75,64.94)	
NH-Black (*n* = 1742)	13.34(11.03,15.65)	10.09(7.99,12.18)	9.14(6.75,11.53)	
NH-Asian (*n* = 954)	5.94(4.60,7.29)	7.67(5.73,9.61)	2.65(2.01,3.29)	
Hispanic (*n* = 690)	7.34(6.13,8.55)	6.79(5.74,7.84)	8.24(6.54,9.93)	
Mexican American (*n* = 962)	6.46(4.78, 8.14)	8.86(6.63,11.08)	13.54(10.51,16.57)	
Other or multiracial (*n* = 378)	4.00(3.33,4.67)	4.20(3.08,5.32)	5.59(4.11,7.06)	
Educational attainment				<0.001
Less than high school graduate (*n* = 1,457)	12.71(11.56,13.87)	12.73(11.40,14.07)	12.05(10.89,13.20)	
High school graduate or GED (*n* = 1815)	53.78(50.39,57.18)	56.43(52.68,60.19)	64.44(62.04,66.83)	
Some college or above (*n* = 3,853)	33.50(29.68,37.33)	30.83(26.77,34.89)	23.52(21.12,25.91)	
Family income–to-poverty ratio				0.081
<1.30 (n = 2094)	20.86(18.80,22.92)	17.87(16.66,19.08)	21.06(18.66,23.45)	
1.30–3.49 (*n* = 2,840)	33.66(31.17,36.15)	37.93(34.22,41.64)	34.97(32.64,37.31)	
≥3.50 (*n* = 2,191)	45.48(42.57,48.39)	44.21(40.41,48.00)	43.97(41.77,46.17)	
Smoking status				<0.001
Never (*n* = 4,245)	62.00(59.44,64.57)	63.04(59.86,66.21)	46.01(43.16,48.87)	
Former (*n* = 1,629)	20.05(18.19,21.90)	27.57(24.76,30.38)	28.89(26.86,30.93)	
Now (*n* = 1,250)	17.95(15.92,19.98)	9.39(7.75,11.03)	25.09(22.62,27.56)	

NH, non-Hispanic; GED, General Educational Development.

### 3.2 Comorbidities and laboratory and physical parameters

In Table 2, comorbidities were more prevalent in individuals with MASLD than in those without MASLD. The glucose metabolism indices were elevated in participants with MASLD relative to those in participants with MetALD. Conversely, liver enzyme levels and blood lipid indices were higher in the MetALD group than in the MASLD group. Individuals without SLD were more likely to engage in high levels of physical activity (78.54%) compared to those with MASLD (68.16%) or MetALD (72.75%). Similarly, the proportion of participants without SLD who adhered to a high-quality diet (HQD) (36.37%) was significantly greater than that observed in individuals with MASLD (30.08%) or MetALD (23.65%).

**Table 2:** Clinical characteristics of participants with non-SLD, MASLD, and MetALD in the NHANES study, United States, 2017–2020 (*n* = 7,125).

	Non-SLD Weighted mean or percentage (95% CI) *n* = 3,025	MASLD Weighted mean or percentage (95% CI) *n* = 2,555	MetALD Weighted mean or percentage (95% CI) *n* = 1,545	*P*-value
Comorbidities				
Prediabetic (*n* = 2,622)	27.87(26.11,29.63)	43.61(40.96,46.26)	42.70(39.95,45.46)	<0.001
Diabetes (*n* = 1,471)	4.83(4.03, 5.62)	26.80(25.08,28.53)	17.80(16.12,19.48)	<0.001
Hypertension (*n* = 3,056)	22.39(20.10, 0.68)	48.29(45.76,50.83)	42.89(39.64,46.15)	<0.001
ASCVD (*n* = 698)	5.34(4.62, 6.06)	11.56(9.92,13.20)	8.33(6.49,10.17)	<0.001
MAFLD (*n* = 2,827)	0.00(0.00, 0.00)	98.04(97.25,98.84)	97.79(96.35,99.22)	<0.001
Anthropometrics				
BMI (kg/m^2^)	25.64(25.30,25.97)	32.87(32.48,33.25)	33.23(32.68,33.78)	<0.001
WC (cm)	89.32(88.46, 90.17)	109.49(108.54,110.45)	109.39(108.18,110.61)	<0.001
Lab panel				
Fasting glucose (mg/dl)	101.82(101.19,102.45)	120.19(118.53,121.86)	113.11(111.72,114.49)	<0.001
Fasting insulin (uU/mL)	10.85(10.26,11.43)	21.07(19.72,22.41)	20.81(19.29,22.33)	<0.001
Glycosylated hemoglobin (%)	5.40(5.38,5.42)	5.98(5.93,6.02)	5.75(5.70,5.80)	<0.001
Alanine aminotransferase (U/L)	19.15(18.53,19.76)	24.31(23.73,24.88)	28.30(27.13,29.47)	<0.001
Aspartate aminotransferase (U/L)	21.13(20.54,21.71)	21.73(21.30,22.17)	24.24(23.47,25.00)	<0.001
Alkaline phosphatase (U/L)	74.05(72.40,75.70)	79.24(78.12,80.36)	79.29(77.85,80.73)	<0.001
Gamma-glutamyltransferase (U/L)	23.04(21.93,24.16)	30.47(29.45,31.49)	40.45(38.40,42.49)	<0.001
Total bilirubin (mg/dl)	0.48(0.47,0.49)	0.47(0.46,0.49)	0.44(0.42,0.46)	0.002
Albumin (g/dl)	4.15(4.13,4.18)	4.07(4.05,4.09)	4.06(4.03,4.09)	<0.001
Triglycerides (mg/dl)	157.60(149.63,165.57)	207.43(193.63,221.23)	212.95(201.59,224.32)	<0.001
Total cholesterol (mg/dl)	182.18(180.27,184.10)	188.59(185.97,191.21)	194.73(191.81,197.65)	<0.001
HDL	58.04(57.47,58.61)	48.50(47.82,49.18)	50.90(49.92,51.88)	<0.001
LDL	93.22(91.69, 94.74)	100.23(98.28,102.18)	102.53(100.55,104.52)	<0.001
HS-CRP (mg/dl)	2.72(2.48,2.97)	4.60(4.34,4.87)	4.63(4.25,5.01)	<0.001
VCTE measurements				
CAP (dB/m)	204.91(203.57,206.24)	308.86(306.69,311.02)	309.92(307.04,312.81)	<0.001
LSM (kPa)	4.94(4.83,5.05)	6.58(6.31,6.84)	6.80(6.41,7.19)	<0.001
PA (min/week)				
Continuous scale	1613.68(1502.53,1724.84)	1185.71(1091.47,1279.96)	1607.65(1456.95,1758.35)	<0.001
*Cutoffs*				<0.001
Low active (*n* = 757)	9.17(7.90,10.45)	19.37(17.38,21.36)	15.69(13.19,18.19)	
Moderate active (*n* = 456)	12.28(10.58,13.98)	12.46(10.94,13.99)	11.57(9.80,13.33)	
High active (*n* = 5,912)	78.54(76.67,80.42)	68.16(65.94,70.39)	72.75(70.23,75.26)	
HEI (2015)				
Continuous scale	50.23(48.46,51.99)	49.02(47.80,50.24)	46.71(44.72,48.71)	0.027
*Tertiles*				0.002
LQD (*n* = 2,375)	31.21(29.40,33.02)	34.69(29.92,39.46)	39.46(32.65,46.27)	
Borderline-quality die (*n* = 2,375)	32.42(29.91,34.93)	35.23(31.30,39.17)	36.90(33.70,40.09)	
HQD (*n* = 2,375)	36.37(33.15,39.59)	30.08(26.68,33.47)	23.65(18.62,28.67)	

ASCVD, atherosclerotic cardiovascular disease; MAFLD, metabolic dysfunction associated fatty liver disease; BMI, body mass index; WC, waist circumference; HDL, high-density lipoprotein; LDL, low-density lipoprotein; HS-CRP, high-sensitive c-reactive protein; VCTE, vibration-controlled transient; CAP, controlled attenuation parameter; LSM, liver stiffness measure; elastography; PA, physical activity; HEI, Healthy Eating Index; LQD, low diet quality; HQD, high diet quality.

### 3.3 Associations of DQ and PA with MASLD and MetALD risks

Table 3 presents the covariate-adjusted associations of DQ, PA, and SES with the risks of MASLD and MetALD. Compared to a LQD, an HQD was associated with significantly lower risks of MASLD (OR: 0.49, 95% CI: 0.38–0.62) and MetALD (OR: 0.45, 95% CI: 0.36–0.56). Similarly, High PA levels were linked to reduced risks of MASLD (OR: 0.47, 95% CI: 0.38–0.58) and MetALD (OR: 0.53, 95% CI: 0.39–0.72). Educational attainment also influenced MASLD risk. Participants with a college degree or higher had a lower risk of MASLD (OR: 0.73, 95% CI: 0.55–0.96, *p* = 0.032) compared to those with less than a high school education. However, this association was not observed for MetALD. Additionally, a higher PIR was associated with a reduced risk of MASLD (OR: 0.78, 95% CI: 0.62–1.00, *p* = 0.048) compared to MetALD.

**Table 3:** Associations of DQ, PA, and SES with risks of MASLD and MetALD (NHANES 2017–2020).

	Crude model				Model 1			
	MASLD		MetALD		MASLD		MetALD	
	OR(95% CI)	*P*-value	OR(95% CI)	*P-*value	OR(95% CI)	*P*-value	OR(95% CI)	*P*-value
Educational attainment								
Less than high school graduate	1 (ref.)	_			1 (ref.)	_	1 (ref.)	
High school graduate or GED	1.04(0.82,1.32)	0.728	1.18(1.02,1.37)	0.024	1.05(0.83,1.34)	0.649	1.23(1.01,1.50)	<0.001
Some college or above	1.03(0.85,1.24)	0.795	0.99(0.86,1.13)	0.869	0.73(0.55,0.96)	0.032	0.94(0.79,1.12)	0.477
Family income to poverty ratio								
<1.30	1 (ref.)	_			1 (ref.)	_	1 (ref.)	_
1.30–3.49	1.43(1.10,1.86)	0.008	0.95(0.72,1.25)	0.709	1.04(0.81,1.34)	0.757	0.94(0.71,1.24)	0.654
≥3.50	1.26(1.04,1.53)	0.020	0.84(0.66,1.07)	0.147	0.78(0.62,1.00)	0.048	0.95(0.74,1.22)	0.671
PA (min/week)								
Continuous scale	1.00(1.00,1.00)	<0.001	0.98(0.97,0.99)	<0.001	1.00(1.00,1.00)	<0.001	0.99(0.98,0.99)	<0.001
Cutoffs								
Low active	1 (ref.)	_	1 (ref.)	_	1 (ref.)	_	1 (ref.)	
Moderate active	0.49(0.43,0.57)	<0.001	0.55(0.38,0.80)	0.003	0.46(0.34,0.62)	<0.001	0.56(0.37,0.86)	0.009
High active	0.41(0.36,0.47)	<0.001	0.54(0.42,0.70)	<0.001	0.47(0.38,0.58)	<0.001	0.53(0.39,0.72)	<0.001
*P* for trend		<0.001		<0.001		<0.001		<0.001
HEI (2015)								
Continuous scale	0.98 (0.98–0.99)	<0.001	0.99(0.98,1.00)	0.002	0.99(0.99,0.99)	<0.001	0.99(0.98,0.99)	<0.001
Tertiles								
LQD	1 (ref.)	_	1 (ref.)	_	1 (ref.)	_	1 (ref.)	_
Borderline-quality diet	0.95(0.86,1.05)	0.267	0.92(0.77,1.09)	0.322	0.73(0.62,0.86)	<0.001	0.84(0.68,1.03)	0.095
HQD	0.82(0.68,1.00)	0.047	0.51(0.43,0.60)	<0.001	0.49(0.38,0.62)	<0.001	0.45(0.36,0.56)	<0.001
*P* for trend		0.047		<0.001		<0.001		<0.001

Crude model: there are no covariates were adjusted.

Model 1: age, gender, race, education level, family income to poverty ratio, smoking status, PA, and HEI.

ref, reference; PA, physical activity; HEI, Health Eating Index.

### 3.4 Combined effects of DQ and PA on MASLD and MetALD risks

**Figure 2:**
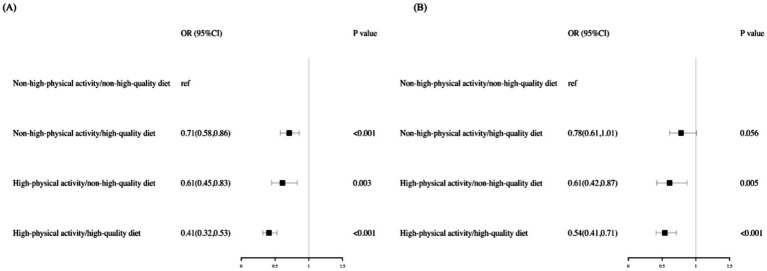
Adjusted risk across the MASLD and MetALD by PA levels and diet quality. Risk of MASLD **(A)** and MetALD **(B)** were computed via logistic regressions models. All analyses were adjusted for age, gender, race, education level, family income to poverty ratio, and smoking status. The reference group are participants with a non–high-quality diet (HEI ≤ 55.67) and non-high-physical activity (<300 min per week). ref., reference; OR, Odds Ratio; CI, confidence interval.

Compared with participants with non-high-physical activity and a non-HQD, high-activity participants with an HQD exhibited the lowest risk for MASLD (OR: 0.41, 95% CI: 0.32–0.53), followed by high-activity participants with a non-HQD (OR: 0.61, 95% CI: 0.45–0.83) and non-high-activity participants with an HQD (OR: 0.71, 95% CI: 0.58–0.86). Compared with that in MASLD, the decreased risk of high-activity participants with an HQD was higher in MetALD (OR: 0.54, 95%CI: 0.41–0.71), followed by high-activity participants with a non-HQD (OR: 0.61, 95% CI: 0.42–0.87) and non-high-activity participants with an HQD (OR: 0.78, 95% CI: 0.61–1.01) (Figure 2).

### 3.5 Subgroups

Examination of the interactions between PA, DQ, and the risks of MASLD and MetALD, stratified by age, sex, race, education level, PIR, and BMI, revealed significant subgroup differences. For MASLD, high PA levels were particularly protective among individuals with a lower BMI compared to those with low PA (*P* for interaction <0.001). Compared to a LQD, participants with higher SES and younger obese individuals exhibited the lowest risk of MASLD with an HQD. For MetALD, physically active individuals with higher SES demonstrated the lowest risk. Additionally, females, individuals with higher SES, and obese participants with an HQD showed a significantly reduced risk of MetALD compared to those with a non-HQD. These results are detailed in Tables 4, 5.

**Table 4:** Stratified associations between physical activity, diet quality, and MASLD risk across various subgroups (NHANES 2017–2020).

	Low active	Moderate active OR(95% CI)	High active OR(95% CI)	*P* for interaction	LQD	Borderline-quality diet OR(95% CI)	HQD OR(95% CI)	*p* for interaction
Age (years)				0.139				0.032
18–34	ref	0.37(0.22,0.61)	0.34(0.16,0.72)		ref	0.71(0.46,1.11)	0.42(0.27,0.64)	
35–49	ref	0.40(0.27,0.59)	0.39(0.22,0.69)		ref	0.65(0.39,1.08)	0.47(0.30,0.75)	
50–64	ref	0.56(0.37,0.83)	0.47(0.29,0.76)		ref	1.08(0.84,1.39)	0.50(0.36,0.71)	
≥65	ref	0.86(0.59,1.25)	0.79(0.48,1.30)		ref	0.88(0.55,1.41)	0.76(0.47,1.23)	
Gender				0.027				0.283
Female	ref	0.36(0.26,0.49)	0.36(0.28,0.47)		ref	1.04(0.83,1.30)	0.81(0.63,1.05)	
Male	ref	0.66(0.43,1.01)	0.38(0.27,0.52)		ref	0.90(0.75,1.08)	0.93(0.71,1.20)	
Race				0.024				<0.001
NH-White	ref	0.38(0.25,0.58)	0.35(0.25,0.50)		ref	0.83(0.73,0.95)	0.64(0.48,0.85)	
NH-Black	ref	1.14(0.69,1.88)	0.76(0.60,0.98)		ref	1.11(0.90,1.38)	0.98(0.69,1.40)	
NH-Asian	ref	0.76(0.60,0.97)	0.73(0.60,0.89)		ref	1.55(1.02,2.37)	1.20(0.74,1.94)	
Mexican American	ref	0.50(0.28,0.89)	0.31(0.22,0.44)		ref	1.34(0.92,1.95)	1.07(0.72,1.60)	
Hispanic	ref	0.88(0.55,1.39)	0.46(0.27,0.76)		ref	0.88(0.51,1.50)	0.83(0.50,1.39)	
Educational attainment				0.074				0.002
Less than high school graduate	ref	1.14(0.66,1.96)	0.62(0.41,0.96)		ref	1.78(1.25,2.54)	1.13(0.72,1.78)	
High school graduate or GED	ref	0.78(0.38,1.58)	0.53(0.33,0.85)		ref	0.98(0.64,1.50)	0.73(0.57,0.95)	
Some college or above	ref	0.36(0.24,0.53)	0.35(0.26,0.46)		ref	0.99(0.80,1.24)	0.70(0.55,0.91)	
Family income–to-poverty ratio				0.209				0.007
<1.30	ref	0.59(0.34,1.01)	0.36(0.28,0.47)		ref	1.14(0.89,1.45)	0.96(0.76,1.22)	
1.30–3.49	ref	0.58(0.37,0.89)	0.56(0.42,0.74)		ref	1.21(0.97,1.52)	1.05(0.81,1.35)	
≥3.50	ref	0.40(0.25,0.63)	0.35(0.23,0.53)		ref	0.75(0.58,0.97)	0.73(0.56,0.94)	
BMI (kg/m^2^)				<0.001				0.034
<25	ref	0.78(0.45,1.36)	0.31(0.20,0.50)		ref	2.05(1.28,3.29)	0.94(0.55,1.60)	
25–30	ref	0.61(0.35,1.08)	0.30(0.22,0.43)		ref	1.15(0.87,1.51)	0.98(0.67,1.44)	
≥30	ref	0.31(0.19,0.50)	0.58(0.39,0.84)		ref	0.99(0.70,1.41)	0.60(0.43,0.84)	

All analyses were adjusted for age, gender, race, education level, family income to poverty ratio, and smoking status. OR, Odds Ratio; CI, confidence interval. ref, reference; OR, Odds Ratio; CI, confidence interval; LQD, low-quality-diet; HQD, high-quality-diet.

**Table 5:** Stratified associations between physical activity, diet quality, and MetALD risk across various subgroups (NHANES 2017–2020).

	Low active	Moderate active OR(95% CI)	High active OR(95% CI)	*P* for interaction	LQD	Borderline-quality diet OR(95% CI)	HQD OR(95% CI)	*p* for interaction
Age (years)				0.159				0.596
18–34	ref	0.60(0.36,1.00)	0.48(0.30,0.76)		ref	0.73(0.49,1.08)	0.44(0.29,0.65)	
35–49	ref	0.42(0.28,0.63)	0.33(0.15,0.70)		ref	0.66(0.44,0.99)	0.32(0.22,0.46)	
50–64	ref	0.87(0.43,1.73)	0.88(0.48,1.62)		ref	1.03(0.59,1.82)	0.48(0.32,0.72)	
≥65	ref	0.68(0.34,1.35)	0.47(0.24,0.91)		ref	0.99(0.46,2.13)	0.58(0.28,1.19)	
Gender				0.347				0.002
Female	ref	0.48(0.34,0.68)	0.47(0.30,0.73)		ref	1.03(0.85,1.25)	0.43(0.34,0.55)	
Male	ref	0.70(0.42,1.17)	0.54(0.40,0.74)		ref	0.84(0.64,1.10)	0.63(0.51,0.79)	
Race				<0.001				<0.001
NH-White	ref	0.47(0.32,0.69)	0.45(0.25,0.79)		ref	0.76(0.58,1.00)	0.35(0.28,0.44)	
NH-Black	ref	1.10(0.75,1.63)	0.80(0.55,1.16)		ref	1.48(1.14,1.92)	1.25(0.83,1.87)	
NH-Asian	ref	5.39(2.05,14.14)	4.55(1.88,11.00)		ref	0.61(0.36,1.03)	0.54(0.26,1.13)	
Mexican American	ref	0.95(0.47,1.90)	0.52(0.30,0.90)		ref	1.20(0.76,1.88)	1.08(0.73,1.60)	
Hispanic	ref	0.49(0.22,1.07)	0.34(0.16,0.72)		ref	1.28(0.79,2.07)	0.89(0.49,1.63)	
Educational attainment				<0.001				<0.001
Less than high school graduate	ref	1.05(0.64,1.73)	0.97(0.68,1.40)		ref	1.73(1.19,2.52)	1.60(1.07,2.39)	
High school graduate or GED	ref	0.64(0.47,0.86)	0.61(0.46,0.80)		ref	0.98(0.65,1.47)	1.01(0.73,1.39)	
Some college or above	ref	1.08(0.61,1.91)	0.38(0.25,0.60)		ref	0.76(0.60,0.96)	0.33(0.26,0.41)	
Family income–to-poverty ratio				0.039				0.002
<1.30	ref	0.89(0.64,1.22)	0.59(0.45,0.78)		ref	1.40(1.03,1.88)	0.79(0.56,1.13)	
1.30–3.49	ref	0.79(0.55,1.12)	0.78(0.58,1.06)		ref	1.08(0.88,1.34)	0.65(0.46,0.92)	
≥3.50	ref	0.66(0.39,1.09)	0.43(0.29,0.64)		ref	0.64(0.47,0.86)	0.34(0.26,0.45)	
BMI (kg/m^2^)				0.069				<0.001
<25	ref	0.85(0.30,2.41)	0.66(0.33,1.35)		ref	2.15(1.11,4.15)	0.88(0.43,1.80)	
25–30	ref	0.81(0.42,1.57)	0.65(0.38,1.11)		ref	1.01(0.68,1.50)	0.61(0.44,0.85)	
≥30	ref	0.57(0.41,0.80)	0.35(0.21,0.57)		ref	0.52(0.36,0.74)	0.50(0.38,0.66)	

All analyses were adjusted for age, gender, race, education level, family income to poverty ratio, and smoking status. OR, Odds Ratio; CI, confidence interval. ref, reference; OR, Odds Ratio; CI, confidence interval; LQD, low-quality-diet; HQD, high-quality-diet.

### 3.6 Associations among PA, DQ levels, and cACLD prevalence

The prevalence of physically active individuals with cACLD was 63.37%, while that of HQD with cACLD was 26.35% (Supplementary Table S3 and Supplementary Figure S1).

### 3.7 Associations of DQ and PA with cACLD risk

Compared to an LQD, HQD was associated with a decreased risk of cACLD (OR: 0.47, 95% CI: 0.31–0.72). Increased PA significantly reduced cACLD risk (OR: 0.45, 95% CI: 0.32–0.63). Both PA and DQ levels were inversely associated with cACLD risk in a non-linear dose–response manner (*P* for trend <0.001). Supplementary Table S4 lists the covariate-adjusted associations of DQ, PA, and SES with cACLD risk.

### 3.8 Combined effects of DQ and PA on cACLD risk

Compared to non-high-active participants with a non-HQD, high-active participants with an HQD displayed the lowest risk for cACLD (OR: 0.44, 95% CI: 0.24–0.82), followed by non-high-active participants with an HQD (OR: 0.57, 95% CI: 0.39–0.81) and high-active participants with a non-HQD (OR: 0.67, 95% CI: 0.47–0.97) (Supplementary Figure S1).

## 4 Discussion

This cross-sectional study explored the associations between PA, DQ, and the prevalence of MASLD and MetALD, as well as their correlation with cACLD in participants from the NHANES 2017–2020 dataset. Our findings reveal a significant inverse relationship between both PA and HQD with the risks of MASLD, MetALD, and cACLD, highlighting the essential role of lifestyle factors in the prevention and progression of liver diseases associated with metabolic dysfunction.

The prevalence rates of MASLD and MetALD in our study were 35.07 and 21.46%, respectively, which differ significantly from those reported in previous studies using the same NHANES 2017–2020 dataset (30, 31). Lee et al. (31) defined MASLD as steatosis (CAP ≥288 dB/m) with at least one metabolic risk factor, with sensitivity analyses at a lower CAP threshold (≥248 dB/m). They defined MetALD as MASLD with an average daily alcohol intake of ≥20 g for women and ≥30 g for men in the past 12 months. In contrast, our study defined MASLD based on the absence of viral hepatitis or significant alcohol consumption, using a CAP threshold of 285 dB/m. Additionally, beyond alcohol consumption, our study included in MetALD patients with steatosis resulting from drugs, monogenic diseases, or other combined etiologies, which differs from other research. These variations in diagnostic criteria and CAP thresholds can lead to significant differences in reported prevalence rates. Despite these differences, the overall patterns observed in our study align with the broader understanding of MASLD and MetALD epidemiology, highlighting the significant burden of these conditions and the critical role of lifestyle factors in their management.

Comorbidities were more prevalent in individuals with MASLD than in those with MetALD. Elevated glucose metabolism indices were observed in participants with MASLD relative to those with MetALD. Conversely, liver enzyme levels and blood lipid indices were higher in the MetALD group than in the MASLD group. These findings suggest that while MASLD is closely associated with metabolic dysfunction, MetALD presents with more severe liver-specific abnormalities. Interestingly, our results contrast with previous research indicating that MetALD is associated with higher rates of metabolic abnormalities and poorer outcomes compared to MASLD (30). Although previous research suggests poorer outcomes in MetALD due to severe metabolic abnormalities and advanced liver damage, our findings highlight that MASLD can also present significant metabolic challenges (32).

Our results also underscore the pivotal role of PA and DQ in reducing the risks of MASLD, MetALD, and cACLD by targeting shared mechanisms, including insulin resistance, inflammation, oxidative stress, liver fat accumulation, and fibrosis. In MASLD, PA improves insulin sensitivity, reduces visceral fat, and enhances lipid metabolism, which helps mitigate hepatic steatosis (33). High levels of PA (≥300 min/week) promote fatty acid oxidation, reduce liver fat, and decrease systemic inflammation—key factors in preventing the progression of steatohepatitis (34). An HQD, rich in fiber, omega-3 fatty acids, and antioxidants, complements PA by reducing oxidative stress and inflammation, improving metabolic health, and preventing fat accumulation in the liver (35). The synergistic effects of PA and HQD help lower liver fat, reduce inflammatory cytokines, and improve insulin sensitivity, all of which are critical in preventing and managing MASLD. In MetALD, where alcohol-induced oxidative stress exacerbates liver damage, PA helps alleviate inflammation and oxidative damage, reducing the harmful effects of alcohol metabolism (36). While PA offers protective antioxidant effects, an HQD enhances these effects by providing vitamins C and E, polyphenols, and omega-3 fatty acids, which neutralize reactive oxygen species (ROS) and reduce liver inflammation (37). Micronutrients such as folate also support alcohol metabolism, helping to mitigate the liver’s response to alcohol-induced damage (38). The combination of PA and HQD thus works synergistically to reduce inflammation, oxidative stress, and metabolic dysfunction, providing significant protection against alcohol-related liver injury. In cACLD, where fibrosis is largely irreversible, PA enhances the resilience of the remaining healthy liver tissue, improves endothelial function, and supports liver regeneration. While PA cannot reverse fibrosis, it helps stabilize liver function and prevent further progression (39). An HQD, which includes protein, vitamins, and minerals, supports liver function and maintains the integrity of the remaining hepatic tissue. Moreover, HQD reduces systemic inflammation and oxidative stress, stabilizes fibrosis, and modulates the gut-liver axis, which is crucial for preventing further disease progression (40, 41). Together, PA and HQD help stabilize cACLD, prevent decompensation, and support liver resilience.

Subgroup analyses revealed that PA levels were particularly protective in individuals with lower BMI, indicating that those with lower BMI may experience greater metabolic benefits from PA, including reductions in visceral fat and enhanced insulin sensitivity (33). For MASLD, participants with higher SES and younger obese individuals exhibited the lowest risk with an HQD, likely due to better access to nutritious foods and greater metabolic flexibility in younger populations (42). In MetALD, physically active individuals with higher SES demonstrated the lowest risk, highlighting the synergistic effects of PA and SES in mitigating alcohol-induced liver damage. Additionally, females, individuals with higher SES, and obese participants with an HQD showed significantly reduced risks of MetALD, suggesting that HQD, which is rich in antioxidants and anti-inflammatory nutrients, may help alleviate the oxidative stress and inflammation caused by alcohol metabolism. These findings emphasize the varying effectiveness of PA and HQD across subgroups, with younger, healthier, and more socioeconomically advantaged populations benefiting the most. Targeted interventions promoting both PA and HQD, particularly in vulnerable groups, could significantly reduce the risks of MASLD and MetALD. In cACLD, the influence of SES is more pronounced, primarily due to better access to resources that foster PA and a wider variety of healthy food options. These advantages are critical for managing obesity and metabolic syndrome, which are key factors in preventing progression to cACLD.

Our study builds on previous research demonstrating an inverse relationship between MAFLD and PA and DQ (43), consistent with studies linking HQD, PA, and higher education with reduced risk of NAFLD (29). Our findings further support the idea that lifestyle modifications, including increased PA and improved DQ, play a central role in reducing the risks of MASLD, MetALD, and cACLD. While cACLD has traditionally been seen as a stage of liver disease where interventions may have limited efficacy, our results suggest that PA and HQD continue to provide significant protective effects at this stage, challenging the notion that lifestyle changes are less effective in advanced disease.

Although our study utilizes NHANES data, which is a nationally representative cohort that enhances the generalizability of our findings, several limitations should be considered. One notable limitation is the exclusion of certain vulnerable subgroups, such as institutionalized individuals, which may affect the representativeness of the sample. As a result, the findings may not fully reflect the experiences or risks of these populations, potentially limiting the broader applicability of the results. Additionally, the cross-sectional design of our study prevents the establishment of causal relationships. While the observed associations suggest potential pathways for intervention, future longitudinal studies are required to confirm causality and assess the long-term effects of physical activity and diet quality on liver disease outcomes. Self-reported data on physical activity and diet quality, although commonly used in large-scale studies, may introduce recall bias, which could impact the accuracy of these measures.

In conclusion, our findings emphasize the critical role of PA and HQD in reducing the risk of liver diseases such as MASLD, MetALD, and cACLD. These results support the importance of lifestyle interventions in liver health and highlight the need for public health strategies and clinical approaches that promote physical activity and improve diet quality to prevent and manage liver diseases. Further research is necessary to better understand the underlying mechanisms and confirm the causality of these relationships through longitudinal studies.

## Data Availability

The datasets presented in this study can be found in online repositories. The names of the repository/repositories and accession number(s) can be found in the article/Supplementary material.
