# Supplementary material for: Diet quality and physical activity affect metabolic dysfunction-associated steatotic liver disease, metabolic dysfunction and etiology-associated steatohepatitis, and compensated advanced chronic liver disease among United States adults: NHANES 2017–2020
Source: Front Nutr. 2025 Jan 8;11:1505970. doi: 10.3389/fnut.2024.1505970 (PMC11750654; doi:10.3389/fnut.2024.1505970)
Supplement: Supplementary file 1 [file Data_Sheet_1.docx]

Table S1 NHANES Variables Related to Diet

| **Component** | **NHANES Variable Name** | **Description** |
| --- | --- | --- |
| Diet | DR1TOT_C | Total energy intake (kcal) |
| Diet | DR1TKCAL | Total kilocalories |
| Diet | DR1T_PROT | Total protein intake (g) |
| Diet | DR1T_FAT | Total fat intake (g) |
| Diet | DR1T_SFAT | Saturated fat intake (g) |
| Diet | DR1T_CARB | Total carbohydrate intake (g) |
| Diet | DR1T_SUGR | Total sugar intake (g) |
| Diet | DR1T_FIBE | Total fiber intake (g) |

Table S2 NHANES Variables Related to Physical Activity

| **Component** | **NHANES Variable Name** | **Description** |
| --- | --- | --- |
| Physical Activity | PAQ605 | Moderate-intensity sports, fitness, or recreational activities in minutes per week |
| Physical Activity | PAQ620 | Vigorous-intensity sports, fitness, or recreational activities in minutes per week |
| Physical Activity | PAQ665 | Work activity level |

Table S3 Prevalence of physical activity and diet quality among cACLD participants (NHANES 2017–2020)

|  | Non-SLD(n=3025) | Non-cACLD (n=3365) | cACLD  (n=735) | P value |
| --- | --- | --- | --- | --- |
| **PA (MET min/week)** | 1613.68(1502.53,1724.84) | 1393.04(1310.69,1475.39) | 1185.72( 984.11,1387.33) | < 0.001 |
| *Cutoffs* |  |  |  |  |
| Low active | 9.17( 7.90,10.45) | 16.91(15.37,18.45) | 25.71(20.97,30.45) |  |
| Moderate active | 12.28(10.58,13.98) | 12.18(10.88,13.49) | 10.92( 7.37,14.48) |  |
| High active | 78.54(76.67,80.42) | 70.91(69.26,72.56) | 63.37(58.01,68.73) |  |
| **HEI (2015) score** |  |  |  |  |
| *Tertiles* |  |  |  | < 0.001 |
| LQD | 31.21(29.40,33.02) | 33.83(31.59,36.07) | 40.99(34.96,47.02) |  |
| Borderline-quality diet | 32.42(29.91,34.93) | 34.75(32.73,36.77) | 32.16(26.37,37.95) |  |
| HQD | 36.37(33.15,39.59) | 31.42(29.28,33.56) | 26.85(22.38,31.33) |  |

Abbreviations:PA, physical activity; HEI, Healthy Eating Index; LQD, low diet quality; HQD, high diet quality.

Table S4 Associations of DQ, PA, and SES with risks of cACLD (NHANES 2017-2020)

|  | Crude Model |  | Model 1 |  |
| --- | --- | --- | --- | --- |
|  | cACLD |  | cACLD |  |
|  | OR(95% CI) | *P* value | OR(95% CI) | *P* value |
| **Educational attainment** |  |  |  |  |
| Less than high school graduate | 1 (ref.) |  | 1 (ref.) |  |
| High school graduate or GED | 1.63(1.25,2.12) | <0.001 | 1.77(1.33,2.36) | <0.001 |
| Some college or above | 0.60(0.38,0.96) | 0.029 | 0.64(0.38,1.09) | 0.085 |
| **Family income to poverty ratio** |  |  |  |  |
| <1.30 | 1 (ref.) |  | 1 (ref.) |  |
| 1.30-3.49 | 1.31(1.08, 1.58) | 0.005 | 1.37(1.01,1.86) | 0.045 |
| ≥3.50 | 0.72(0.55,0.93) | 0.015 | 0.67(0.50,0.90 | 0.005 |
| **PA (min/week)** |  |  |  |  |
| Continuous scale | 0.99 (0.99-0.99) | <0.001 | 0.99 (0.99-0.99) | <0.001 |
| *Cutoffs* |  |  |  |  |
| Low active | 1 (ref.) | _ | 1 (ref.) | _ |
| Moderate active | 0.46(0.28,0.75) | 0.002 | 0.46(0.26,0.80) | 0.005 |
| High active | 0.43(0.32,0.59) | <0.001 | 0.45(0.32,0.63) | <0.001 |
| ***p* for trend** |  | <0.001 |  | <0.001 |
| **HEI (2015)** |  |  |  |  |
| Continuous scale | 0.98 (0.98-0.99) | 0.002 | 0.98(0.97,0.99) | 0.001 |
| *Tertiles* |  |  |  |  |
| LQD | 1 (ref.) | _ | 1 (ref.) | _ |
| Borderline-quality die | 0.84(0.64,1.11) | 0.208 | 0.89(0.68,1.18) | 0.414 |
| HQD | 0.42(0.28,0.64) | 0.004 | 0.47(0.31,0.72) | <0.001 |
| ***p* for trend** |  | <0.001 |  | <0.001 |

Note: Ordinal logistic regression model

Crude Model: There are no covariates were adjusted.

Model 1: age, gender, race, education level, family income to poverty ratio, smoking status, PA, and HEI score.

Abbreviation: ref, reference; PA, physical activity; HEI, Health Eating Index


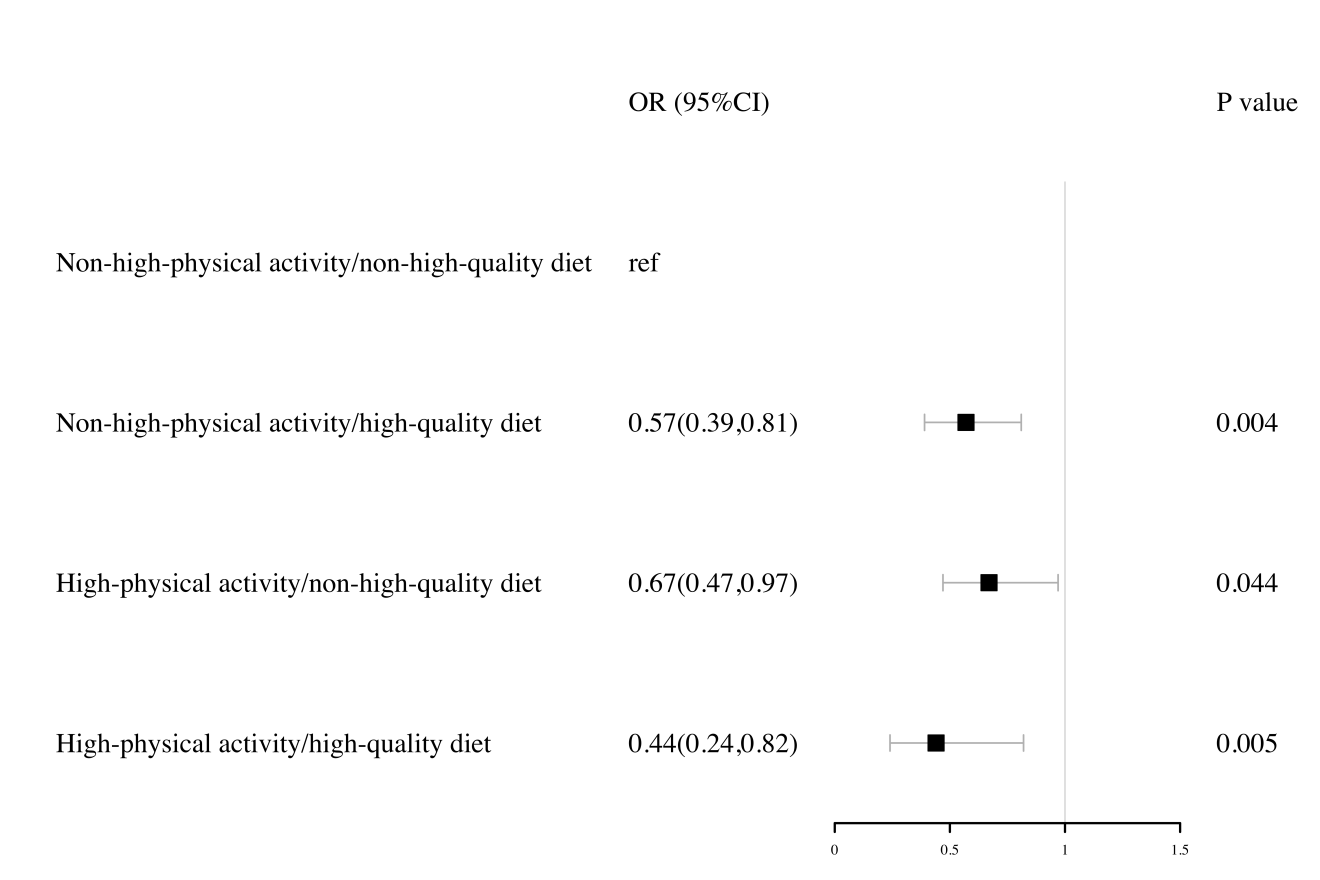


Figure S1. Adjusted risk across the cACLD by PA levels and diet quality. Risk of cACLD was computed via ordinal logistic regressions models. All analyses were adjusted for age, gender, race, education level, family income to poverty ratio, and smoking status. The reference group are participants with a non–high-quality diet (HEI ≤ 55.67) and non-high-physical activity (<300 minutes per week). Abbreviation: ref, reference; OR, Odds Ratio; CI, confidence interval.
